# The Association Between Self-Managed versus Clinician-Managed Abortion and Self-Reported Abortion Complications: A Cross-Sectional Analysis in India

**DOI:** 10.2147/IJWH.S414599

**Published:** 2023-09-28

**Authors:** Sophie Goemans, Abhishek Singh, Ajit Kumar Yadav, Lotus McDougal, Anita Raj, Sarah H Averbach

**Affiliations:** 1 Department of Obstetrics, Gynecology, and Reproductive Sciences, University of California San Diego, La Jolla, CA, USA; 2 Department of Public Health & Mortality Studies, International Institute for Population Sciences, Mumbai, Maharashtra, India; 3 Centre of Demography of Gender, International Institute for Population Sciences, Mumbai, Maharashtra, India; 4 GENDER Project, International Institute for Population Sciences, Mumbai, Maharashtra, India; 5 Center on Gender Equity and Health, University of California San Diego, La Jolla, CA, USA

**Keywords:** reproductive autonomy, reproductive choice, self-use, medication abortion, misoprostol, mifepristone

## Abstract

**Purpose:**

To examine the association between self-managed abortion and the self-reported experience of abortion complications in India, a country with a high incidence of self-managed abortion.

**Patients and Methods:**

The study used a cross-sectional multivariable logistic regression analysis of data from the National Family Health Survey (NFHS-4) of 2015–2016 to compare the odds of self-reported complications experienced during abortion between self-managed and clinician-managed abortions in India.

**Results:**

On average, self-managed abortions occurred earlier in gestation than clinician-managed abortions, 7.8 weeks and 11.3 weeks, respectively (p < 0.001). Self-managed abortion was associated with fewer self-reported abortion-related complications than clinician-managed abortions when adjusted for covariates not including gestational age (Adjusted Odds Ratio (aOR) 0.82, 95% confidence interval (CI) 0.69, 0.97). However, once adjusted for gestational age, there was no longer a clinically meaningful or statistically significant difference in the odds of self-reported complications between self-managed and clinician-managed abortions (aOR = 0.98, 95% CI 0.81, 1.18).

**Conclusion:**

These findings suggest that people in India are using safe methods to self-manage abortions and support the hypothesis that self-managed abortion can improve access to abortion and reproductive choice without increasing risk.

## Introduction

Abortion has been legal in India since 1971.^1^ In India, a quarter of abortions are reported to be self-managed, that is, managed by the pregnant person without consultation from a licensed clinician.^2^ The legality of self-managed abortion, in Indian law, is not explicitly addressed; however, medication abortion was legalized in 2002 and requires a prescription.^1^,^3^ In the past, methods of self-managed abortion included herbal remedies, blunt force or inserting instruments into the uterus, all of which have lower efficacy and higher risk than clinician managed abortion.^2^,^4^ However, with the increasing availability of safe and effective medication abortion regimens (misoprostol with or without mifepristone), it is believed that self-managed abortion has shifted to self-use of medication abortion.^2^,^5^,^6^

Provided that people access accurate information and correct medications and dosages for self-use medication abortion, self-managed abortion can be as safe as clinician-managed abortion.^2^,^4^,^7^,^8^ Safe and effective self-use medication abortion has the potential to increase access to abortion in places where it is legally restricted, or where clinician managed abortion is limited, and/or improve reproductive autonomy in areas where abortion is not restricted.^9^

Though self-managed abortion is common in India, it is unknown how often people experience complications with self-managed abortion compared to clinician-managed abortion. Assuming that accurate information and correctly dosed high-quality abortion medications are not widely accessible throughout India without a prescription, we hypothesized that self-managed abortion would have higher complication rates than clinician-managed abortion.^5^ We performed a cross-sectional analysis of a large, nationally representative survey to gain a better understanding of the association between self-reported complications during self-managed and clinician-managed abortion in India.

## Materials and Methods

This is a cross-sectional analysis of the association between self-managed abortion versus clinician-managed abortion and self-reported abortion-related complications among a nationally representative cohort of reproductive aged women (15–49 years old) in India. Data was collected from the National Family Health Survey (NFHS-4) of 2015–16. The NFHS-4 version was chosen for analysis because previous versions of this survey addressed other women’s health topics but did not explicitly elicit information about who performed an abortion or abortion complications; subsequent enumerations were not publicly available at the time of the analysis. Of the 699,686 female respondents, 8251 women reported an abortion in the five previous years. The survey was administered by the International Institute of Population Sciences (IIPS); data were collected by female interviewers in the preferred language of the participant.

We defined self-managed abortion (SMA) as a self-reported abortion performed by or supervised by the pregnant person, a friend, a family member, or Dai (traditional birth attendant). We defined clinician-managed abortion (CMA) as a self-reported abortion performed by or supervised by a licensed clinician (doctor, nurse, or auxiliary nurse midwife). The type of abortion (self-managed vs clinician-managed) and the reported abortion location (home, clinic, hospital, other/elsewhere) were compared to validate these definitions. The locations were grouped into clinic-type locations (public or private hospital or clinic) and other locations (at home or other/elsewhere). We defined a self-reported abortion-related complication as a “yes” answer to the question “Did you have any complication from the abortion?” We included the following covariates in the analysis: age of the pregnant person, parity, having a living son, caste (upper vs lower), completed education, wealth quintile, residence (urban or rural), and gestational age of the pregnancy. Gestational age at the time of abortion was asked in months; these data were subsequently converted to weeks through multiplication by a factor of 4.34 (average number of weeks in a month) for ease of results interpretation. All covariates were chosen a priori as potential confounders of the association between self-managed abortion and self-reported abortion-related complications and were identified using a directed acyclic graph.^10^

Bivariate comparisons were tested using chi-squared tests. Logistic regression models were used to assess the relationship between self-managed abortion and self-reported abortion-related complications, including unadjusted bivariable regressions, and two multivariable models. The first multivariable model included age, parity, having a living son, belonging to a “scheduled caste, scheduled tribe or other backward caste” (governmentally recognized and defined categorizations of social marginalization in India), education, wealth quintile^11^ (as represented by a principal components analysis of household assets and housing characteristics), and residence (urban/rural). The second multivariable model included all previous covariates and gestational age.

Analyses were conducted using STATA (version 15.1).

All participants provided verbal informed consent. Ethical approval for data collection was provided by the Institutional Review Board of International Institute of Population Sciences and ICF. Ethical approval for analysis of the de-identified dataset was approved by the Institutional Review Board of the University of California, San Diego.

## Results

Of the 699,686 female respondents, 8251 women reported an abortion in the five previous years (1.2%). Of the 8251 pregnancies ending in abortion, 2418 (28%) were reported as self-managed, and 2111 (87%) of self-managed abortions were reported to have occurred outside of clinics (data not shown in tables). Of the 5833 clinician-managed abortion, 5709 (98%) were reported to have occurred in clinics (data not shown in tables). There were no significant differences in demographic characteristics between those having self-managed abortion and those having clinician-managed abortions, other than gestational age and level of completed education (Table 1). Abortions with complications occurred on average at a later gestational age (9.9 weeks [9.5, 10.4]) than abortions without complications (8.1 weeks [7.9, 8.3]). Those with lower levels of completed education had higher rates of abortion complications (Table 1).

**Table 1:** Descriptive Analysis of Those Aged 15–49, Who Responded to the National Family Health Survey-4 of 2015–16 Reporting an Abortion in the Previous 5 Years (N = 8251). SC = Scheduled Caste, ST = Scheduled Tribe, OBC = Other Backwards Caste, as Defined by the Constitution of India

	Complication (N = 1497)	Without Complication (N = 6754)	
**Mean Gestational Age at Termination**	9.9 weeks [9.5, 10.4]^a^	8.1 weeks [7.9, 8.3]^b^	*p < 0.05
**Abortion type**			p = 0.07
Clinician-managed	1082 (75%)	4751 (72%)	
Self-managed	415 (25%)	2003 (28%)	
**Mean Birth Parity**	2.18 [2.06, 2.30]	2.07 [2.02, 2.12]	p > 0.05
**Living Son**			p = 0.53
No	483 (32%)	1889 (31%)	
Yes	1014 (68%)	4865 (69%)	
**Mean Age of Woman**	28.66 years [28.25, 29.07]	28.39 years [28.19, 29.59]	p > 0.05
**Caste**			p = 0.66
Not SC/ST or OBC	439 (30%)	2093 (31%)	
SC/ST or OBC	1058 (70%)	4661 (69%)	
**Completed Education**			*p < 0.05
No education	332 (22%)	1358 (19%)	
Primary	224 (15%)	818 (12%)	
Secondary	750 (51%)	3718 (54%)	
Higher	191 (13%)	860 (14%)	
**Wealth Quintile**			p = 0.17
1st (lowest)	226 (15%)	904 (13%)	
2nd	310 (20%)	1416 (18%)	
3rd	345 (22%)	1579 (21%)	
4th	327 (22%)	1515 (25%)	
5th (highest)	289 (21%)	1340 (23%)	
**Residence**			p = 0.12
Rural	1007 (62%)	4466 (58%)	
Urban	490 (38%)	2288 (41%)	

**Notes**: *Significant at p<0.05. ^a^Converted from months: mean 2.75 months (95% CI 2.64–2.85). ^b^Converted from months through multiplication by a factor of 4.34 (average number of weeks in a month): mean 2.33 months (95% CI 2.28–2.38).

Self-managed abortions occurred on average at a significantly earlier gestational age for SMA than for CMA (7.8 weeks [7.7, 8.2] for SMA, 11.3 weeks [11.1, 11.6] for CMA, p < 0.001) (data not shown in tables).

After adjusting for age, parity, having a living son, caste, education, wealth quintile and residence, self-managed abortion was associated with fewer self-reported abortion-related complications than clinician-managed abortions (Adjusted Odds Ratio (aOR) 0.82, 95% confidence interval (CI) 0.69, 0.97) (Table 2). There was no longer a statistically significant difference after adjusting for gestational age at the time of abortion (aOR = 0.98, 95% CI 0.81, 1.18) (Table 2).

**Table 2:** Logistic Regressions Assessing the Relationship Between Type of Abortion (Self-Managed Abortion Vs Clinician-Managed Abortion) and Abortion Complication. Model 1 Includes Bivariable Logistic Regressions. Model 2 Has Covariates of Age, Parity, Having a Living Son, Caste, Education, Income, and Residence. Model 3 Has All Previous Covariates and Gestational Age (N = 8251). SC = Scheduled Caste, ST = Scheduled Tribe, OBC = Other Backwards Caste, as Defined by the Constitution of India

	Model 1: Bivariable Regressions	Model 2: Multivariable Regressions without Gestational age	Model 3: Multivariable Regressions with Gestational Age
	Unadjusted Odds Ratio of Complication [95% CI]	Adjusted Odds Ratio of Complication [95% CI]	Adjusted Odds Ratio of Complication [95% CI]
**Mean Gestational Age at Termination**	1.23 [1.16, 1.30]	N/A	1.23 [1.15, 1.31]
**Abortion type**			
Clinician-managed	REF	REF	REF
Self-managed	0.85 [0.71, 1.01]	0.82* [0.69, 0.97]	0.98 [0.81, 1.18]
**Mean Birth Parity**	1.04 [0.99, 1.10]	1.03 [0.96, 1.10]	1.04 [0.97, 1.11]
**Living Son**			
No	REF	REF	REF
Yes	0.94 [0.79, 1.13]	0.86 [0.71, 1.05]	0.94 [0.77, 1.15]
**Mean Age of Woman**	1.01 [0.99, 1.02]	1.00 [0.99, 1.02]	1.01 [0.99, 1.02]
**Caste**			
Not SC/ST or OBC	REF	REF	REF
SC/ST or OBC	1.05 [0.86, 1.27]	1.00 [0.81, 1.21]	0.97 [0.79, 1.18]
**Completed Education**			
No education	REF	REF	REF
Primary	1.00 [0.77, 1.30]	1.05 [0.80, 1.37]	1.09 [0.83, 1.42]
Secondary	0.80* [0.65, 0.97]	0.88 [0.69, 1.11]	0.94 [0.75, 1.20]
Higher	0.76 [0.58, 1.00]	0.84 [0.61, 1.16]	0.95 [0.69, 1.32]
**Wealth Index**			
1st (lowest)	REF	REF	REF
2nd	0.92 [0.71, 1.18]	0.95 [0.73, 1.24]	0.97 [0.75, 1.27]
3rd	0.87 [0.68, 1.11]	0.93 [0.71, 1.22]	0.96 [0.73, 1.27]
4th	0.75* [0.58, 0.97]	0.83 [0.61, 1.13]	0.87 [0.64, 1.19]
5th (highest)	0.76 [0.58, 1.00]	0.87 [0.61, 1.25]	0.92 [0.64, 1.31]
**Residence**			
Rural	REF	REF	REF
Urban	0.87 [0.73, 1.04]	0.93 [0.75, 1.16]	0.97 [0.78, 1.20]

**Note**: *Significant at p < 0.05.

## Discussion

We found that approximately one in four abortions in India are self-managed. Our findings suggest that on average, people in India access self-managed abortion at an earlier gestational age than clinician-managed abortion. Globally, people access self-managed abortion for various reasons – some do so by choice and others by necessity.^2^ However, studies have found that barriers to clinical care are often cited as reasons to pursue self-managed abortion.^2^ Barriers to accessing clinical care, such as cost, access to transportation, legal abortion restrictions, or an available qualified clinician, can delay or even prevent access to clinician managed abortion. People may face fewer barriers to accessing self-sourced medication abortion resulting in people accessing self-managed abortion earlier than clinician-managed abortion.

Contrary to our hypothesis, this study found that self-managed abortion in India is associated with lower odds of self-reported abortion-related complications. While this is not in line with some existing state-level research,^12^ our nationally representative study indicates that overall, Indian women who self-managed abortions tend to have fewer self-reported complications from those abortions. This suggests that on average, women may be utilizing safe methods for self-managing abortion, such as self-use of medication abortion accompanied by correct dosing of quality medications and information about safe use of these medications.^6^ Indeed, the use of medication abortion for self-managed abortion has steadily increased in India since the approval of medication abortion in 2005, through over-the-counter sales by pharmacists and providers outside of the formal clinical setting.^13^ A study in Madhya Pradesh found that only 13.8% of pharmacists asked for a prescription when asked about procuring medication abortion suggesting that the medications may be more routinely available without a prescription.^14^

We found that self-managed abortions occur on average, at an earlier gestation. Increasing gestational age is known to be a risk factor for abortion complications.^15^ When we adjusted for gestational age, self-managed abortion and clinician-managed abortion there was no longer a statistically or clinically significant difference in the odds of complications– suggesting that women who self-manage abortions have lower complication rates overall, not necessarily because the methods used are safer than clinician managed abortion, but rather, because they are able to access abortion care at an earlier gestational age. We found similar complication rates for self-managed and clinician-managed abortions after adjusting for gestational age, which suggests that self-managed abortion is a safe approach for increasing access to abortion and reproductive choice in India. It is also possible, however, that women preferentially seek abortion care in a facility when they know they are further in their pregnancy because of concern for potential complications rather than barriers to acquiring facility-based care delaying their abortion. A qualitative evaluation of why women self-manage their abortion or to seek abortion care in a facility in India would add value.

Self-managed abortion with medication abortion can be a safe method of accessing abortion care when correctly dosed, affordable, and using quality medications with accurate, accessible administration information.^16^ Medication abortion is increasingly available throughout India without prescription. However, while prior studies have highlighted the challenges of accessing correctly dosed medications and accurate information, this research has been limited to select Indian states.^12^,^17^,^18^ Our study provides additional data about the experience of self-reported complications among women who have a self-managed versus clinician-managed abortion in a nationally representative dataset.

Strengths of our study include a nationally representative dataset which offers an opportunity to assess for self-managed abortion and compare self-reported outcomes between self-managed abortion and clinician-managed abortion. In addition, our dataset is limited to recent abortions (experienced from 2010 to 2016) reflecting more contemporary availability of medication abortion.

Our findings should be considered in light of several study limitations. The percent of women reporting an abortion in the last 5 years (1.2%) was lower than other studies in India have estimated (4% per year).^6^ The data were based on self-reported information and were subject to social desirability bias, therefore abortions and self-managed abortions are likely being underreported. Additionally, any abortion-related deaths were not included since the dataset captures only self-reported outcomes. Unfortunately, the type and severity of the reported complications were not asked and should be further studied. Gestational age may also be differently reported between groups due to differences in dating methods. Dating for clinician-managed pregnancies is more likely to be confirmed by clinicians through ultrasonography. However, this does not suggest there will be discordance in dating – one study in Nepal showed a high level of agreement between reported last menstrual period and bimanual dating in an abortion seeking population.^19^ Furthermore, the cross-sectional design of the study prevents us from establishing a causal relationship between self-managed abortion and complications.

While the self-reported complication rates were similar between groups, we found higher rates of complications in both groups than studies which ascertain clinical outcomes and cite an adverse event rate ranging from 1.3% to 8.1%.^2^ Our measure of ‘abortion-related complications’ was self-reported and likely included known rare adverse events (such as hemorrhage, receipt of antibiotics, need for surgical intervention) but also expected side-effects (such as pain, heavy bleeding, or self-limiting low-grade fever), and signs of potential complications that did not lead to adverse events (such as vaginal discharge, persistent pain, heavy vaginal bleeding, or need to visit a health facility). Therefore, complications, as we have measured them here, likely include normal side effects and visits to health facilities for confirmation of abortion completion. However, this measure is patient-centered and reflects the patient experience of abortion. In addition, it is useful as a measure despite the heterogeneity in what is being measured because we were able to compare this outcome between self-managed and clinician-managed abortions.

## Conclusion

We found that self-managed abortion and clinician-managed abortion had comparable rates of self-reported complications after adjusting for gestational age, suggesting that people are accessing accurate information, medication dosage and quality medications to self-manage abortions.^16^ Overall, this supports self-managed abortion as a safe method to increase access to abortion and reproductive choice for people in India. Access to clinical care remains important for post-abortion care and to address concerns or adverse events, and to provide clinically supervised medication abortion and procedural abortion for those who prefer this option or have a contraindication to medication abortion.
